# Frozen embryo transfer outcomes in patients with prior cesarean delivery: a focus on endometrial lining parameters

**DOI:** 10.1016/j.xfre.2025.12.006

**Published:** 2026-01-02

**Authors:** Emily A. Clarke, Jing Wu, Hannah Milad, Kerry Flannagan, Jiarui Wang, Joseph Lee, Luis Hoyos, Meike Uhler, Michael Homer, Jenna Friedenthal, Alan B. Copperman, Phillip Romanski, Mary Morris

**Affiliations:** a Department of Obstetrics, Gynecology, and Reproductive Science, Icahn School of Medicine at Mount Sinai, New York, New York; b US Fertility, Rockville, Maryland; c Loyola University Medical Center, Maywood, Illinois; d Reproductive Medicine Associates of New York, New York; e IVF Florida Reproductive Associates, Margate, Florida; f Fertility Centers of Illinois, Chicago, Illinois; g Reproductive Science Center, San Francisco, California; h Shady Grove Fertility, Towson, Maryland

**Keywords:** Cesarean delivery, frozen embryo transfer, endometrial thickness, endometrial receptivity, live birth rates

## Abstract

**Objective:**

To evaluate the association between prior mode of delivery (cesarean vs. vaginal) and endometrial thickness in the subsequent frozen embryo transfer (FET) cycle. The secondary aim was to evaluate FET cycle outcomes.

**Design:**

Retrospective cohort study.

**Subjects:**

This multicenter study included patients with one previous live birth who initiated a subsequent, autologous FET cycle from January 2012 to December 2024. Patients were grouped by mode of prior delivery: cesarean or vaginal.

**Exposure:**

Cesarean or vaginal delivery.

**Main Outcome Measures:**

The primary outcome was endometrial thickness at final lining assessment. Secondary outcomes included FET cycle cancellation rate due to thin or fluid-filled endometrium, pregnancy, and live birth rates. Subgroup analyses evaluated outcomes of euploid FET cycles only.

**Results:**

A total of 11,131 FET cycles consisting of 5,149 from patients with prior cesarean and 5,982 from patients with prior vaginal delivery were included. Endometrial thickness in the subsequent FET cycle was similar between cohorts (cesarean: 10.4 mm [10.3–10.5 mm]; vaginal: 10.4 mm [10.3–10.4 mm]). The FET cycle cancellation rate due to thin endometrium was comparable (cesarean: 1.3%, vaginal: 1.2%; adjusted relative risk [aRR] 1.02 [0.72–1.43]); cancellation due to endometrial fluid accumulation was higher in the cesarean cohort (1.0% vs. 0.5%; aRR 2.22 [1.40–3.58]). Cesarean delivery was associated with lower live birth (49.5% vs. 53.0%; aRR 0.95 [0.90–0.98]) and clinical pregnancy (60.5% vs. 64.1%; aRR 0.95 [0.90–0.99]), with similar rates of clinical pregnancy loss (cesarean: 10.6%, vaginal: 10.6%; aRR 0.95 [0.84–1.07]). Similar trends were observed in patients undergoing euploid FET, with lower live birth demonstrated in those with a prior cesarean (55.3% vs. 60.3%; aRR 0.91 [0.84–0.98]), but similar chance of clinical pregnancy loss (cesarean: 9.1%, vaginal: 9.4%; aRR 0.95 [0.77–1.17]).

**Conclusion:**

Although patients can be reassured that a prior cesarean does not appear to be associated with reduced endometrial thickness, receptivity in FET cycles may be diminished, as reflected by lower subsequent live birth rates. One possible explanation is an altered endometrial environment, given the increased risk of cycle cancellation due to endometrial fluid accumulation in patients with prior cesarean delivery.

Cesarean delivery is the most common uterine surgery performed worldwide, with rates rising over the last 50 years to 32% of live births in the United States (1, 2). Although often a necessary obstetric intervention, cesarean delivery is known to increase risks of complications in future pregnancies, including abnormal placentation, uterine rupture, pelvic adhesions, and potential reduction in fertility (1, 2). Patients with a prior cesarean delivery represent a substantial portion of those undergoing assisted reproductive technology treatment. Patients with a history of assisted reproductive technology-conceived pregnancies returning for a subsequent embryo transfer may have a higher incidence of a prior cesarean section compared with the general population, as pregnancies conceived via in vitro fertilization (IVF) are associated with 1.90-fold increased odds of resulting in a cesarean delivery (3). Although some studies report conflicting findings (4, 5, 6), growing evidence suggests that patients with prior cesarean delivery who undergo IVF have lower implantation and live birth rates compared with those with a history of vaginal delivery (7, 8, 9, 10, 11). However, the mechanisms underlying reduced embryo transfer success in patients with a prior cesarean remain unclear. Proposed mechanisms include the formation of postoperative adhesions or a cesarean scar niche (isthmocele), which may contribute to endometrial fluid accumulation, intrauterine inflammation, or fibrosis of the endometrium (12, 13, 14).

Several studies have shown that uterine surgeries such as dilation and curettage and myomectomy may influence endometrial thinning (15, 16, 17, 18, 19). A thin endometrial lining before frozen embryo transfer (FET) has consistently been associated with inferior IVF outcomes, including lower implantation and live birth and a higher risk of early pregnancy loss (20, 21, 22, 23, 24). Despite the high incidence of cesarean delivery in patients undergoing IVF, few studies have evaluated whether a history of cesarean delivery contributes to reduced endometrial thickness during FET cycles.

Two recent studies found that patients with prior cesarean delivery had comparable endometrial thickness before FET compared with those with a prior vaginal delivery (6, 7). However, neither of those studies was designed to evaluate endometrial thickness as a primary outcome, nor did they account for cycles canceled due to thin or fluid-filled endometrium. The influence of prior cesarean delivery on subsequent endometrial thickness is therefore not fully elucidated. Additionally, it is uncertain whether patients with prior cesarean delivery are more likely to experience FET cycle cancellation due to thin endometrial lining or fluid-filled endometrium. This study, therefore, aims to assess endometrial thickness during FET cycles and evaluate the risk of cycle cancellation due to these factors in patients with a prior cesarean compared with those with a prior vaginal delivery.

## Materials and methods

### Participants and study design

This multicenter, retrospective cohort study included patients with a previous live birth who initiated a subsequent autologous single embryo FET cycle from January 2012 to December 2024. Patients were grouped by mode of prior delivery: cesarean or vaginal. Only the first FET cycle after the first live birth was evaluated. Exclusion criteria included: interval pregnancy or fresh embryo transfer between the first live birth and subsequent FET, use of a gestational carrier, day 3 embryo transfer, a diagnosis of uterine factor infertility before the initial live birth, or initial live birth achieved via spontaneous conception. Patients with uncorrected hydrosalpinx were also excluded. The FET cycles canceled before the first ultrasound assessment or within 9 days of the FET cycle start were excluded, because these patients lacked the opportunity to demonstrate endometrial development.

Demographic data, initial live birth outcomes, and variables from the subsequent FET cycle were collected from the electronic medical record at each study site. Demographic data included patient age (at the start of both the initial live birth and subsequent FET cycle), body mass index (BMI) (kg/m^2^) at FET, gravidity at the start of subsequent FET cycle, primary infertility diagnosis, serum antimüllerian hormone concentration (ng/mL) at the start of the initial live birth cycle, and number of embryo transfers undertaken before the cycle that achieved initial live birth. Initial live birth data included the type of conception (ovulation induction, IVF with fresh embryo transfer, or IVF with FET), gestational age at delivery (weeks), number of live births (singleton or multiple gestation), and mode of delivery (cesarean or vaginal). Mode of delivery was obtained by patient report, with additional obstetric outcomes, including postpartum hemorrhage and chorioamnionitis, not obtained. Subsequent FET cycle variables included time interval between live birth and FET (months), oocyte age (patient age at oocyte retrieval), use of preimplantation genetic testing for aneuploidy (PGT-A), endometrial preparation protocol (natural or programmed), and endometrial thickness (mm) at final lining assessment, day of blastocyst development at cryopreservation (day 5, 6, or 7), and embryo grade. Blastocysts were evaluated by embryologists at their institution using the Gardner grading system; they were then assigned a grade according to the simplified Society for Assisted Reproductive Technology grading system (good, fair, poor) (25). The ease of embryo transfer, as documented by the physician (easy, moderate, or difficult), was also obtained.

The reason for FET cycle cancellation was collected and categorized as due to thin endometrium, fluid accumulation within the endometrial lining, or unrelated to the endometrium. For cycles canceled due to thin endometrium, endometrial thickness on the day of cancellation was assessed. The study was approved by the Institutional Review Boards at each participating study center.

### Endometrial thickness evaluation

Endometrial thickness was assessed at the final lining assessment. Final lining assessment was considered the last ultrasound before progesterone initiation in programmed FET cycles, or before luteinizing hormone (LH) surge or trigger shot administration in natural FET cycles. Endometrial thickness was measured at the maximum distance (mm) between the echogenic interface of the myometrium and endometrium, in a midsagittal ultrasonographic view of the uterus.

### Procedures

Controlled ovarian hyperstimulation protocols were chosen by the treating physician based on age and ovarian reserve. Patients used either an antagonist protocol or a short or long gonadotropin-releasing hormone agonist protocol. Ovarian follicular growth was monitored by transvaginal ultrasound. When two or more follicles reached a mean diameter of 18 mm or greater, a trigger shot of either compounded human chorionic gonadotropin (hCG), gonadotropin-releasing hormone agonist, or both was administered for final oocyte maturation. Ultrasound-guided vaginal oocyte retrieval was then performed 36 hours after trigger administration. Metaphase II oocytes were fertilized using conventional insemination or intracytoplasmic sperm injection. Embryos were then cultured until they reached the blastocyst stage appropriate for biopsy and/or cryopreservation, as previously described (26). For patients using PGT-A, trophectoderm biopsy was performed on day 5, 6, or 7 of embryo development; biopsied samples were cryopreserved by vitrification and sent for PGT-A testing (26). For patients who did not use PGT-A, blastocysts were cryopreserved by vitrification (27).

Before initiating an embryo transfer cycle, all patients underwent uterine evaluation by saline-infused sonogram as per clinic guidelines, with the uterine cavity deemed appropriate for transfer by their physician. Embryo transfer cycles used either programmed or natural endometrial preparation as previously described (28, 29). For programmed endometrial preparation, the endometrium was prepared with estrogen, including micronized oral estradiol, vaginal, dermal, intramuscular, and/or subcutaneous estradiol, for approximately 9 days before assessment of the endometrium by transvaginal ultrasound. When adequate endometrial thickness was achieved, with a goal of at least 7 mm or greater, progesterone was initiated with intramuscular, vaginal, or oral administration. The FET was performed on the 6th day of progesterone administration. Natural FET cycles followed either a natural or modified natural cycle protocol where patients underwent transvaginal ultrasound monitoring of ovarian follicular growth and endometrial lining development, as well as serum hormonal measurements to detect LH surge or determine timing of the hCG trigger administration. After the LH surge or trigger, progesterone was started for luteal support, followed by FET (28). In some natural cycles, exogenous gonadotropins or oral ovulation induction agents were administered to stimulate follicular development. The FET cycle cancellation was at the discretion of the treating physician, based on thin endometrium or fluid accumulation within the endometrial lining at final lining assessment, or due to other factors not related to the endometrium. Embryos were warmed using the standard warming process (27), and embryo transfer was performed under standard conditions as previously described (26).

### Outcomes

Among noncanceled FET cycles, the primary outcome was endometrial thickness at final lining assessment. Secondary outcomes included positive pregnancy (positive serum β-hCG per embryo transfer), clinical pregnancy (presence of intrauterine gestational sac(s) on transvaginal ultrasound assessment per embryo transfer), live birth (live birth per embryo transfer), biochemical loss (pregnancy loss after positive serum β-hCG per embryo transfer), clinical pregnancy loss (pregnancy loss after presence of gestational sac on transvaginal ultrasound assessment per embryo transfer), and ectopic pregnancy (ectopic pregnancy per embryo transfer).

Additional outcomes included the risk of FET cycle cancellation due to thin endometrium or fluid accumulation. Cancellation was calculated as the number of FET cycles canceled for either thin endometrium or fluid accumulation by all initiated FET cycles included in the analysis. At the study sites, FET cycle cancellation is typically considered when endometrial thickness does not surpass <7 mm.

Subgroup analyses of FET cycle outcomes were performed on patients who used PGT-A and transferred a euploid embryo. Additional subgroup analysis was also performed on the primary outcome of endometrial thickness in patients who underwent programmed or natural endometrial preparation.

### Statistical analysis

Statistical analyses were performed using R software (Version 4.4.1; R Foundation for Statistical Computing). Patient demographics, FET cycle parameters including endometrial thickness, FET cancellation rate, and FET outcomes were compared between groups. Continuous data were reported as means with standard deviations, with differences assessed by the Wilcoxon rank-sum test. Categorical data were reported as proportions. Univariate analysis to compare FET cycle cancellation rate and FET outcomes between groups was performed with χ^2^ or Fisher’s exact tests. Multivariable generalized linear models with log-transformed values were used to compare endometrial thickness between groups. Average endometrial thickness values were estimated by back-transforming the log-adjusted model results, with 95% confidence intervals (CIs). Convergence issues were encountered when using a generalized linear model with binomial regression and log-link function to estimate relative risks (RRs), particularly due to the low cancellation rate in this cohort. To address this, multivariable generalized linear models with Poisson regression were conducted to evaluate the associations of FET cycle cancellation and subsequent FET outcomes with prior modes of delivery, with estimations of RR with 95% CIs (30). Prior vaginal delivery served as the reference group. The model for FET cycle cancellation was adjusted for patient age, BMI, and fertility center; the model for subsequent FET outcomes was additionally adjusted for embryo quality (good, fair, poor) (25). A two-sided *P* value of <.05 was considered significant for all analyses.

## Results

A total of 11,131 FET cycles consisting of 5,149 from patients with a prior cesarean delivery and 5,982 from patients with a prior vaginal delivery were included. The overall FET cancellation rate was 4.4% (n = 487), with n = 249 in the cesarean group and n = 238 in the vaginal delivery group. Among the remaining 10,644 FET cycles, 4,900 were in the prior cesarean group and 5,744 in the prior vaginal delivery group.

Patient demographics and FET cycle characteristics were similar in both groups (Table 1). The age of patients was statistically different but clinically similar between groups, both at oocyte retrieval (cesarean: 33.8 ± 3.9 years, vaginal: 32.8 ± 3.7 years, *P*<.01) and at FET (cesarean: 36.5 ± 3.9 years, vaginal: 35.4 ± 3.7 years, *P*<.01), as was BMI at FET (cesarean: 26.7 ± 5.7 kg/m^2^, vaginal: 25.2 ± 5.1 kg/m^2^, *P*<.01). Although also reaching statistical significance (*P*<.01), primary infertility diagnosis was similar between cohorts, with similar proportions of patients with male factor infertility (cesarean: 27.7%, vaginal: 29.9%), unexplained infertility (cesarean: 12.7%, vaginal: 11.3%), and ovulatory dysfunction (cesarean: 18.5%, vaginal: 19.6%). Patients in the prior cesarean cohort underwent more embryo transfers before the cycle that achieved live birth compared with patients in the vaginal delivery cohort (0.6 ± 1.0 vs. 0.5 ± 0.9 transfers, *P*<.01). Patients in both groups waited a similar amount of time after live birth to initiate an FET cycle (cesarean: 23.4 ± 14.3 months, vaginal: 22.4 ± 13.4 months). Most FET cycles in both groups used a programmed endometrial preparation (cesarean: 91.1% of cycles, vaginal: 89.8% of cycles) and transferred a day 5 blastocyst (cesarean: 58.0% of cycles, vaginal: 61.6% of cycles). The overall breakdown of good, fair, and poor-quality blastocysts was clinically similar in both groups. The PGT-A was more commonly used in the prior cesarean compared with the prior vaginal delivery cohort (41.0% vs. 34.0% of cycles, *P*<.01). The vaginal delivery cohort had more “easy” (90.3% vs. 87.3%) and fewer “moderate” (1.0% vs. 4.7%) embryo transfers compared with the prior cesarean cohort (*P*<.01). In both cohorts, the prior live births had been mostly achieved by IVF with FET (cesarean: 61.8%, vaginal: 50.2% of initial live births), followed by IVF with fresh embryo transfer (cesarean: 35.5%, vaginal: 46.7% of initial live births). The remaining pregnancies were achieved by ovulation induction / intrauterine insemination (cesarean: 2.7%, vaginal: 3.1%), with no spontaneous pregnancies. The majority of initial live births were singleton gestations (cesarean: 92.2%, vaginal: 98.4%).

**Table 1:** Demographic and frozen embryo transfer cycle characteristics in patients with prior cesarean or vaginal delivery.

	Cesarean n = 5,149	Vaginal n = 5,982	*P* value
Age at oocyte retrieval (y)	33.8 ± 3.9	32.8 ± 3.7	<.01
Age at FET (y)	36.5 ± 3.9	35.4 ± 3.7	<.01
BMI at FET (kg/m^2^)	26.7 ± 5.7	25.2 ± 5.1	<.01
Gravidity	1.7 ± 1.1	1.6 ± 1.0	<.01
Parity	1.0 ± 0.0	1.0 ± 0.0	.80
AMH (ng/mL)	4.2 ± 4.7	4.4 ± 5.2	.01
Infertility diagnosis (n, %)			<.01
Diminished ovarian reserve	472 (9.2)	436 (7.9)	
Endometriosis	245 (4.8)	236 (4.0)	
Male factor	1,428 (27.7)	1,788 (29.9)	
Ovulatory dysfunction	950 (18.5)	1,169 (19.6)	
Tubal factor	401 (8.0)	399 (6.7)	
Unexplained	991 (19.3)	1,271 (21.3)	
Other/not entered	650 (12.7)	680 (11.3)	
No. of embryo transfers before achieving the initial live birth	0.6 ± 1.0	0.5 ± 0.9	<.01
Initial live birth to FET (mo)	23.4 ± 14.3	22.4 ± 13.4	<.01
Endometrial preparation (n, %)			.04
Programmed	4,693 (91.1)	5,371 (89.8)	
Natural	456 (8.9)	611 (10.2)	
PGT-A tested embryo (n, %)^a^	2,007 (41.0)	1,957 (34.0)	<.01
Blastocyst development at cryopreservation (n, %)^a^			<.01
Day 5	2,841 (58.0)	3,538 (61.6)	
Day 6	1,939 (39.6)	2,109 (36.7)	
Day 7	120 (2.4)	97 (1.7)	
Embryo quality (n, %)^a^			.01
Good	3,309 (67.5)	4,002 (69.7)	
Fair	1,377 (28.1)	1,554 (27.1)	
Poor	142 (2.9)	128 (2.2)	
Not available	72 (1.5)	60 (1.0)	
Ease of embryo transfer (n, %)^a^			<.01
Easy	4,280 (87.3)	5,189 (90.3)	
Moderate	230 (4.7)	59 (1.0)	
Difficult	25 (0.5)	16 (0.3)	
Not available	365 (7.4)	480 (8.4)	

*Note:* Values are mean ± standard deviation or number of patients with (percentages). AMH = antimüllerian hormone; BMI = body mass index; FET = frozen embryo transfer; n = number of patients who initiated FET cycle in each cohort; PGT-A = preimplantation genetic testing for aneuploidy.

a Indicates percentage of blastocysts transferred in noncanceled cycles: Cesarean, n = 4,900; Vaginal, n = 5,744.

Endometrial thickness at final lining assessment before FET was similar between evaluated cohorts: 10.7 ± 2.3 mm in the prior cesarean delivery cohort and 10.7 ± 2.2 mm in the prior vaginal delivery cohort (*P*=.40) (Table 2). After adjusting for age, BMI, and fertility center, endometrial thickness remained statistically similar: 10.4 mm [95% CI 10.3–10.5] in the cesarean delivery cohort and 10.4 mm [95% CI 10.3–10.4 mm] in the vaginal delivery cohort (*P*=.35). In subgroup analysis of patients who underwent programmed vs. natural endometrial preparation, after adjusting for covariates, similar trends were observed: endometrial thickness was similar between mode of delivery groups in both the programmed cycle subgroup (cesarean: 10.4 mm [95% CI 10.4–10.5], vaginal: 10.4 mm [95% CI 10.3–10.5], *P*=.56) and natural cycle subgroup (cesarean: 10.0 mm [95% CI 9.7–10.3], vaginal: 9.9 mm [95% CI 9.5–10.2], *P*=.17).

**Table 2:** Endometrial thickness and frozen embryo transfer cycle cancellation rates due to thin endometrium or endometrial fluid accumulation.

	Cesarean n = 5,149	Vaginal n = 5,982	Cesarean adjusted [95% CI]	Vaginal adjusted [95% CI]	*P* value
Endometrial thickness (mm)^a^	10.7 ± 2.3	10.7 ± 2.2	10.4 [10.3–10.5]	10.4 [10.3–10.45]	.35
Cancellation due to thin endometrium (n, %)	67 (1.3)	73 (1.2)	1.02 [0.72–1.43]	*Reference*	.91
Cancellation due to endometrial fluid accumulation (n, %)	51 (1.0)	30 (0.5)	2.22 [1.40–3.58]	*Reference*	<.01

*Note:* Values are means ± standard deviation, or number of patients with (percentages). Relative risk was adjusted for patient age, BMI, and fertility center, with prior vaginal delivery as the reference group. BMI = body mass index; CI = confidence interval; FET = frozen embryo transfer; n = number of patients who initiated frozen embryo transfer in each cohort.

a Endometrial thickness in patients who underwent FET: Cesarean, n = 4,900; Vaginal, n = 5,744.

The FET cycle cancellation rate due to thin endometrium was similar between groups (cesarean: 1.3%, vaginal: 1.2%, *P*=.70; adjusted RR [aRR] 1.02 [95% CI 0.72–1.43]), with 67 of 5,149 initiated FET cycles canceled in the prior cesarean delivery cohort compared with 73 of 5,982 initiated FET cycles canceled in the prior vaginal delivery cohort (Table 2). Cancellations due to fluid accumulation was higher in the cesarean delivery cohort compared with vaginal delivery cohort (1.0% vs. 0.5%, *P*<.01; aRR 2.22 [95% CI 1.40–3.58]), with 51 of 5,149 initiated FET cycles and 30 of 5,982 initiated FET cycles canceled, respectively. Overall FET cycle cancellation due to an issue with the endometrial lining (either thin or fluid) was higher in the prior cesarean delivery cohort, at 2.3% compared with 1.7% (*P*<.05), with aRR 1.34 (95% CI 1.02–1.77).

The FET cycle outcomes are displayed in Table 3. Patients with a prior cesarean delivery had a lower live birth rate compared with those with a prior vaginal delivery (49.5% vs. 53.0%, *P*<.01; aRR 0.95 [95% CI 0.90–0.98]). Rates of positive pregnancy and clinical pregnancy were also lower in the cesarean group across both unadjusted and adjusted analyses. However, rates of biochemical pregnancy loss, clinical pregnancy loss, and ectopic pregnancy per transfer were similar between cohorts.

**Table 3:** Frozen embryo transfer outcomes among patients with prior cesarean or vaginal delivery.

	Cesarean n = 4,900	Vaginal n = 5,744	Adjusted RR [95% CI]	*P* value
Positive pregnancy rate (n, %)	3,386 (69.1)	4,208 (73.3)	0.94 [0.90–0.99]	.02
Clinical pregnancy rate (n, %)	2,963 (60.5)	3,683 (64.1)	0.95 [0.90–0.98]	.03
Live birth rate (n, %)	2,444 (49.9)	3,044 (53.0)	0.95 [0.90–0.98]	.04
Biochemical loss rate (n, %)	391 (8.0)	502 (8.7)	0.91 [0.79–1.04]	.16
Clinical loss rate (n, %)	519 (10.6)	519 (10.6)	0.95 [0.84–1.07]	.39
Ectopic pregnancy rate (n, %)	32 (0.7)	23 (0.4)	1.63 [0.94–2.87]	.09

*Note:* Values are number of patients with (percentages). Relative risk was adjusted for patient age, BMI, embryo quality, and fertility center, with prior vaginal delivery as the reference group. BMI = body mass index; CI = confidence interval; n = number of patients who underwent frozen embryo transfer in each cohort; RR = relative risk.

Among patients who underwent FET with PGT-A tested euploid embryo, clinical pregnancy and live birth rates were lower in the cesarean delivery group (Table 4). Live birth per transfer was 55.3% in the cesarean delivery cohort compared with 60.3% in the vaginal delivery cohort (*P*<.01) (aRR 0.91 [95% CI 0.84–0.98]). Rates of biochemical and clinical loss remained similar between groups.

**Table 4:** Single euploid embryo transfer outcomes among patients with prior cesarean or vaginal delivery.

	Cesarean n = 1,989	Vaginal n = 1,938	Adjusted RR [95% CI]	*P* value
Positive pregnancy (n, %)	1,463 (72.9)	1,512 (77.3)	0.94 [0.87–1.01]	.11
Clinical pregnancy (n, %)	1,292 (64.4)	1,363 (69.6)	0.92 [0.85–0.99]	.03
Live birth (n, %)	1,109 (55.3)	1,180 (60.3)	0.91 [0.84–0.98]	.04
Biochemical loss (n, %)	160 (8.0)	140 (7.2)	1.15 [0.91–1.46]	.23
Clinical loss (n, %)	183 (9.1)	183 (9.4)	0.95 [0.77–1.17]	.64
Ectopic (n, %)	11 (0.5)	9 (0.5)	1.23 [0.49–3.11]	.66

*Note:* Values are the number of patients with (percentages). Relative risk adjusted for patient age, BMI, embryo quality, and fertility center, with prior vaginal delivery as the reference group. BMI = body mass index; CI = confidence interval; n = number of patients who underwent frozen embryo transfer in each cohort; RR = relative risk.

## Discussion

Patients with a prior cesarean delivery undergoing a subsequent FET cycle demonstrated similar endometrial thickness compared with those with a prior vaginal delivery. Additionally, both cohorts had a mean endometrial thickness of >10 mm, a range previously associated with favorable FET outcome compared with a thin endometrium lining of <7 mm (20, 21, 22, 23, 24). Similar FET cycle cancellation rates due to a thin endometrium lining between cohorts suggest that prior cesarean delivery does not appear to compromise endometrial development in IVF patients. However, patients with a prior cesarean delivery demonstrated a higher risk of FET cycle cancellation due to fluid accumulation within the endometrial lining, with aRR of 2.22 (95% CI 1.40–3.58).

Findings from this study are consistent with two prior studies that assessed endometrial thickness in FET cycles after cesarean or vaginal delivery, both of which reported no difference between groups (6, 7). However, those studies were not designed to evaluate endometrial thickness as a primary outcome and did not specify the timing of its measurement. In contrast, our study used endometrial thickness at the final lining assessment as the most clinically useful metric to guide progesterone initiation, determine timing of FET, and assess cycle viability. By standardizing the measurement of endometrial thickness, this study was more generalizable across other clinical settings.

More importantly, neither of the previous studies evaluated FET cycles that were canceled due to endometrial lining issues. By excluding these cases, those studies make conclusions that reflect a population inherently biased toward patients who achieved adequate endometrial thickness. To our knowledge, this is the first study to assess FET cycle cancellation due to thin endometrium or fluid within the endometrial lining in patients with a prior cesarean compared with vaginal delivery. Our findings describe both comparable endometrial thickness and similar cancellation rates due to thin endometrium lining between patients with prior cesarean and vaginal delivery, further supporting that a prior cesarean delivery does not appear to influence endometrial thinning in IVF populations.

However, FET cycles were more likely to be canceled due to fluid accumulation within the lining in the prior cesarean compared with prior vaginal delivery cohort. Intracavitary fluid accumulation is most seen in the setting of a cesarean scar niche, or isthmocele, and has been shown to be associated with inferior FET outcomes (6, 7, 13, 31). Given the large size of our study, we could not assess for the ultrasonographic presence of isthmocele in the prior cesarean delivery cohort. Previous studies suggest that more than half of patients with a prior cesarean develop an isthmocele visualized on ultrasound (32), and patients with isthmocele are at a 40% risk of developing intracavitary fluid accumulation during IVF stimulation (33). Further study is needed to elucidate FET outcomes and endometrial development with and without isthmocele visualized on ultrasound in patients with prior cesarean delivery.

Consistent with prior studies, a prior cesarean delivery was associated with lower clinical pregnancy and live birth compared with a prior vaginal delivery (7, 8, 9, 10, 11). Although chances of positive pregnancy, clinical pregnancy, and live birth rates were slightly lower in the cesarean delivery cohort, biochemical loss and clinical pregnancy loss rates were similar between groups. These trends were also observed in the subgroup of patients who underwent single, euploid FET. The euploid subgroup analysis more clearly isolates the potential influence of prior cesarean delivery on subsequent FET outcomes, because implantation failure and pregnancy loss in this context are less likely to be attributed to embryonic aneuploidy (34). The mechanism behind reduced observed reproductive potential in the prior cesarean delivery cohort remains unclear; however, based on findings from this study, it does not appear to be related to endometrial thickness. One possible hypothesis may be in part due to an altered endometrial microbiome or environment (35, 36), because patients with prior cesarean delivery demonstrated an increased risk of FET cycle cancellation due to the presence of endometrial fluid accumulation. Another hypothesis could be the presence of a uterine scar leading to reduced uterine contractility (37, 38), possibly affecting implantation and live birth potential. Further study examining the presence vs. absence of a postoperative isthmocele, in addition to the indication for cesarean delivery and other peripartum complications, would further elucidate potential mechanisms for this observed reduced reproductive potential.

Strengths of this study include its robust study size, its inclusion of canceled cycles and evaluation of cancellation due to thin endometrium and fluid within the endometrial lining, the use of FET cycles only, the subgroup analysis of euploid embryo transfers, and its multicenter design, leading to increased generalizability. However, this study is not without limitations. First, although endometrial thickness is a standard ultrasonographic measurement, there is inherent interobserver bias that cannot be controlled. Further, endometrial thickness before the index live birth was not available, given variety of fertility treatment types used to achieve the initial live birth. Other limitations include its retrospective design, and the inability to stratify by indication for cesarean or other peripartum complications, including chorioamnionitis, postpartum hemorrhage. Further, although all patients underwent a satisfactory uterine cavity evaluation by their physician before initiating the subsequent FET cycle, patients’ interim history between live birth and subsequent FET (cesarean: 23.4 ± 14.3 months, vaginal: 22.4 ± 13.4 months) was not evaluated, including the possible need for operative hysteroscopy for interim abnormal uterine pathology. Further, at participating centers, the presence of an isthmocele is not typically documented in the electronic medical record; therefore, the study was not able to assess for the presence of an isthmocele visualized on ultrasound. However, before initiating an FET cycle, all patients underwent uterine cavity evaluation, with the uterine cavity deemed appropriate for embryo transfer. Finally, the study did not assess the effect of more than one prior cesarean or vaginal delivery. Further research in this population stratified by cesarean indication, number of prior deliveries, and the presence of isthmocele is warranted to further understand the relationship between previous mode of delivery, subsequent endometrial thickness, and subsequent FET outcomes.

## Conclusion

In conclusion, patients with a prior cesarean delivery who underwent a subsequent FET cycle had similar endometrial thickness at final assessment and comparable FET cancellation rates due to thin endometrial lining, relative to those with a prior vaginal delivery. However, patients with a prior cesarean experienced a higher rate of cancellation due to fluid accumulation within the endometrial lining. Consistent with prior studies, patients with a history of cesarean delivery compared with those with a prior vaginal delivery had lower clinical pregnancy and live birth rates, despite similar rates of pregnancy loss. Therefore, although patients can be reassured that a prior cesarean delivery does not appear to impair endometrial thickness, it may be associated with other endometrial changes, such as fluid accumulation, that offer plausibility to reduced implantation potential and live birth rates. Future study is needed to better understand the underlying mechanisms by which a prior cesarean delivery may compromise endometrial function and contribute to inferior FET outcomes compared with prior vaginal delivery.

## Declaration of Interests

E.A.C. has nothing to disclose. J.W. has nothing to disclose. H.M. has nothing to disclose. K.F. has nothing to disclose. J.W. has nothing to disclose. J.L. has nothing to disclose. L.H. has nothing to disclose. M.U. has nothing to disclose. M.H. has nothing to disclose. J.F. has nothing to disclose. A.B.C. has nothing to disclose. P.R. has nothing to disclose. M.M. has nothing to disclose.
